# Ancient genomic evidence reveals dual migration routes and yak introgression history of taurine cattle on the Tibetan Plateau

**DOI:** 10.1093/nsr/nwag201

**Published:** 2026-03-31

**Authors:** Shungang Chen, Jiawen Hou, Yu Gao, Guilian Sheng, Ying Zhang, Shargan Wangdue, Zeba Duoji, Songmei Hu, Qiaomei Fu, Xinyi Liu, Ningbo Chen, Fahu Chen

**Affiliations:** Key Laboratory of Western China’s Environmental Systems (Ministry of Education), College of Earth and Environmental Sciences, Lanzhou University, China; Alpine Paleoecology and Human Adaptation Group (ALPHA), State Key Laboratory of Tibetan Plateau Earth System, Environment and Resources (TPESER), Institute of Tibetan Plateau Research, Chinese Academy of Sciences, China; Key Laboratory of Genetic Evolution & Animal Models, Kunming Institute of Zoology, Chinese Academy of Sciences, China; Yunnan Key Laboratory of Integrative Anthropology, Kunming Institute of Zoology, Chinese Academy of Sciences, China; Alpine Paleoecology and Human Adaptation Group (ALPHA), State Key Laboratory of Tibetan Plateau Earth System, Environment and Resources (TPESER), Institute of Tibetan Plateau Research, Chinese Academy of Sciences, China; School of Environmental Studies, China University of Geosciences (Wuhan), China; State Key Laboratory of Geomicrobiology and Environmental Changes, China University of Geosciences (Wuhan), China; National Centre for Archaeology, China; Tibetan Institute of the Preservation of Cultural Relics, China; Changdu Institute of the Preservation of Cultural Relics, China; Key Laboratory of Archaeological Sciences and Technology (Ministry of Education), Shandong University, China; Institute of Cultural Heritage, Shandong University, China; Key Laboratory of Vertebrate Evolution and Human Origins, Institute of Vertebrate Paleontology and Paleoanthropology, Chinese Academy of Sciences, China; University of the Chinese Academy of Sciences, China; Department of Anthropology, Washington University in St. Louis, USA; Key Laboratory of Animal Genetics, Breeding and Reproduction of Shaanxi Province, College of Animal Science and Technology, Northwest A&F University, China; Key Laboratory of Western China’s Environmental Systems (Ministry of Education), College of Earth and Environmental Sciences, Lanzhou University, China; Alpine Paleoecology and Human Adaptation Group (ALPHA), State Key Laboratory of Tibetan Plateau Earth System, Environment and Resources (TPESER), Institute of Tibetan Plateau Research, Chinese Academy of Sciences, China

Cattle, including taurine (*Bos taurus taurus*) and indicine (*B. t. indicus*) cattle, were independently domesticated in the Fertile Crescent (∼10 500 years before present, a BP) and the Indus Valley (∼8500 a BP), respectively [1,2]. As a vital component of the early farming economy, cattle provided humans with meat, milk, leather and other material resources. They were commonly used as draft animals for transportation and provided manure to enrich soils for the cultivation of soils in agropastoral systems. The domestication processes of cattle and post-domestication dispersals driven by human migrations, featuring distinct geographical ranges of the two lineages of taurine and indicine cattle, have shaped ancient economies across Eurasian and African continents, with profound implications that can still be felt today [3].

The oldest record of domestic cattle in ancient China has been documented at Houtaomuga in Da’an, Jilin Province, dating to ∼5500–5300 calibrated (cal) a BP, as informed by archeological and ancient DNA (aDNA) evidence [4]. The introduction of taurine cattle into the Yellow River region ∼5000 a BP and their arrival on the northeastern Tibetan Plateau ∼3750 a BP are supported by genetic evidence [4,5]. Furthermore, taurine cattle arrived on the southern Tibetan Plateau ∼2500 a BP according to evidence from the

Bangga site in Qonggyai County, Shannan city, southern Tibet, China [6]. Recent archaeogenetic studies further support the introduction of taurine cattle into the Yellow River region ∼5000 a BP and into the northeastern Tibetan Plateau ∼3750 a BP [4,5]. Taurine cattle further spread to the southern Tibetan Plateau ∼2500 a BP, as documented at Bangga and affirmed by both morphological and genetic evidence [6]. It is generally believed that hybridization between historically introduced taurine cattle and indigenous yak (*B. grunniens*), whether human-mediated or occurring naturally, facilitated their adaptation to the high-elevation alpine environment [7]. Such a process could have occurred within the context of existing domesticated yak populations or could have inspired yak domestication, which has played, and continues to play, significant roles in the Tibetan economy.

Despite these recent developments, paleogenomic data of cattle with direct radiocarbon measurements are still scarce on the Tibetan Plateau, with only two archeological sites in the northeastern (Tawendaliha and Changning) and one in the southern (Bangga) plateau producing credible datasets [4–6]. Owing to the deficiency in archaeogenomic research in regions beyond the northeastern and southern parts of the plateau, it is now timely to shift the

research focus to the western and eastern regions of the Tibetan Plateau that are underdeveloped thus far to gain deeper insights into the chronology of the early dispersal of taurine cattle and their interactions with yak on the Tibetan Plateau.

To address this research gap, one *Bos* bone remain (KR14_4k) excavated from the Karuo site, one *Bos* tooth remain (ZGM1_2k) and one *Bos* bone remain (ZGM2_3.2k) excavated from the Gepa Serul cemetery were sampled (Fig. 1a and Fig. S1). Karuo (97°2′E, 31°1′N, 3100 m above sea level (a.s.l.)) is a prehistoric occupation site in Changdu city, eastern Tibet, China, which can be dated to 4800–2800 cal a BP [8]. Gepa Serul (79°49′E, 31°34′N, 3780 m a.s.l.) is a two-phase cemetery in Zanda County, Ngari Prefecture, western Tibet, China that can be dated to 3600–2100 cal a BP [9]. The ^14^C-dating results (Fig. S2) reveal that these three remains date from 4290–4010 cal a BP (KR14_4k), 2305–2006 cal a BP (ZGM1_2k) and 3361–3176 cal a BP (ZGM2_3.2k), respectively.

**Figure 1.**
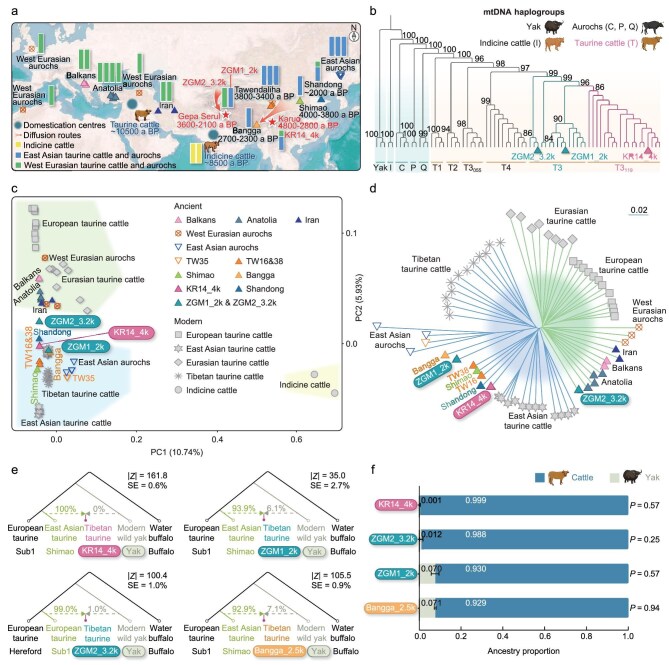
Geographic locations of the analysed ancient cattle and auroch specimens, and results of the genetic analyses performed in this study. (a) Geographic map showing the locations of ancient cattle and auroch specimens involved in genetic analyses, as well as the two possible routes for the early introduction of taurine cattle onto the Tibetan Plateau. The ancestry component assignment of cattle and aurochs was estimated by using NGSadmix with *K* = 3. (b) Maximum likelihood tree of the mitogenomes of yak, aurochs, taurine and indicine cattle. The tree was rooted in a mitogenome of buffalo. The numbers indicate the maximum likelihood of node support after 1000 bootstrap replicates. (c) PCA results for aurochs, taurine and indicine cattle using ANGSD software. The shaded blocks represent different bovine groups. (d) Neighbor-joining tree of aurochs and domestic cattle based on the IBS values. (e) Proportion of yak ancestry in KR14_4k, ZGM1_2k, ZGM2_3.2k and Bangga cattle calculated by using *f*_4_ ratios. (f) Genetic ancestry of KR14_4k, ZGM1_2k, ZGM2_3.2k and Bangga cattle estimated by using qpAdm.

We constructed double-stranded DNA libraries in the aDNA-dedicated laboratory at the China University of Geoscience (Wuhan) to retrieve ancient genomes of these three samples. We subsequently generated shotgun sequencing data with endogenous DNA contents of between 5% and 25% (Table S1). The terminal damage patterns and high degree of fragmentation (Fig. S3) indicated the typical molecular characteristics of aDNA. Genetic identification of these three specimens was first achieved through mitochondrial genome (mitogenome) reconstruction at sequencing depths ranging from 65.06× to 560.70× (Table S1). To determine their species affiliation, we analysed these three mitogenomes together with the known matrilineal variability of cattle, aurochs and yak (Table S2). A maximum likelihood tree assigned ZGM1_2k and ZGM2_3.2k to haplogroup T3 and KR14_4k to sub-haplogroup T3_119_ of taurine cattle (Fig. 1b), which is one of the endemic maternal haplogroups of East Asian taurine cattle.

To further elucidate the whole-genome ancestry of these three individuals, we mapped the shotgun sequencing data to the cattle reference genome (ARS-UCD1.2). We obtained sequencing depths of 0.35–1.07× for the three samples (Table S1). We identified ZGM1_2k and ZGM2_3.2k as males and KR14_4k as a female (Fig. S4). These three ancient genomes were combined with published whole-genome re-sequencing data from contemporary cattle, yak and buffalo (*n* = 59; Table S3) as well as from ancient cattle, aurochs and yak (*n* = 21; Table S4) to investigate their genetic relationships (Table S5). The results of principal component analysis (PCA) and the identity-by-state (IBS) neighbor-joining tree (Fig. 1c and d) reconstruction of autosomal single-nucleotide polymorphisms were consistent with those of the mitogenome assignments, which in turn confirmed that all three samples were genetically related to taurine cattle. KR14_4k, which was unearthed from the Karuo site and dated to 4000 cal a BP, represents the earliest radiocarbon-dated and DNA-confirmed domestic cattle on the Tibetan Plateau thus far. Together with aDNA identification of ZGM2_3.2k and ZGM1_2k unearthed from the Gepa Serul cemetery, these findings suggest that domestic cattle were introduced onto the eastern part of the Tibetan Plateau earlier than 4000 a BP. No later than 3200 a BP, domestic cattle arrived on the western Tibetan Plateau.

Notably, in the PCA plot (Fig. 1c), ZGM1_2k and KR14_4k fall into a cluster that includes ancient cattle from China (e.g. the Shimao, Bangga, Shandong and Tawendaliha sites), along with modern East Asian taurine cattle. These results reveal that ZGM1_2k and KR14_4k were genetically similar to East Asian taurine ancestry. However, ZGM2_3.2k, which was unearthed from the same archeological site but older than ZGM1_2k, was separated from other modern and ancient East Asian taurine cattle, and fell into the transition zone of European and East Asian taurine cattle (Fig. 1c). A neighbor-joining tree based on the IBS values reveals that ZGM1_2k was clustered with ancient Bangga cattle and that KR14_4k was clustered with ancient Shimao, Shandong and Tawendaliha cattle (Fig. 1d). In contrast, ZGM2_3.2k was clustered with ancient European taurine cattle, indicating close genetic affinities with these cattle (Fig. 1d).

Furthermore, the results of admixture analysis (*K* = 2–4) reveal that European taurine cattle and East Asian taurine cattle possess different ancestries. ZGM1_2k and KR14_4k were genetically similar to modern and ancient East Asian taurine cattle (Fig. 1a and Fig. S5). In comparison, ZGM2_3.2k was similar to modern and ancient European taurine cattle (Fig. 1a and Fig. S5). In addition, no ancestry of indicine cattle was detected in these three ancient cattle (Fig. 1a and Fig. S5). *D* statistics were also used to further test the genetic differences between ZGM2_3.2k and other ancient East Asian taurine cattle (ZGM2_3.2k, other ancient East Asian taurine cattle; ancient European taurine cattle, buffalo). The results reveal that, compared with ancient ZGM1_2k, Shimao, Bangga, Tawendaliha and Karuo cattle, ZGM2_3.2k shared more genetic drift with ancient European taurine cattle (Fig. S6 and Table S6). Additionally, qpGraph analysis (Fig. S7) indicates that ZGM2_3.2k exhibited closer genetic affinity to ancient European taurine cattle than to ancient Shimao cattle.

Collectively, these results of nuclear genome analyses suggest that ZGM2_3.2k was genetically different from other ancient East Asian taurine cattle. This sample had a close genetic relationship with European taurine cattle, indicating that, in addition to the known northeastern route [6], there may have been another entrance route for early taurine cattle into the western Tibetan Plateau.

The Tibetan Plateau is more often regarded as a barrier to expansion than are the Inner Asian mountain-corridor or steppe routes. However, the Gepa Serul cemetery has produced the earliest evidence of barley in Tibet (∼3500 a BP), indicating a route of crop diffusion to western Tibet via the Kashgar region of southern Xinjiang or an upward spread from the Indus Valley using Himalayan river valleys [9]. This finding suggests that the Ngari Plateau was connected to a wider world through the Asian mountain regions. Here, we considered an analogous route for the dispersal of domestic cattle to the Ngari Plateau ∼3200 a BP, supporting the mountain-corridor hypothesis (Fig. 1a).

The Indus Valley has always been the main distribution area for indicine cattle [1]. However, admixture analysis reveals no ancestry of indicine cattle in ZGM2_3.2k (Fig. 1a and Fig. S5), which implies that early taurine cattle on the western Tibetan Plateau may not have come from the Indus Valley. In contrast, implementation of the mountain-corridor route, which considers that taurine cattle traveled from Xinjiang to Tibet through the eastern Pamirs, would have required better adaptability of the taurine cattle for alpine regions to spread to higher elevations. This route is similar to National Highway 219, which is also named the Xinjiang–Tibet line. Although the conditions along this road are extremely harsh, with an average altitude of >4500 m a.s.l., it is believed that connections between Xinjiang and Tibet through this path started as early as 3500 a BP [9]. A recently published aDNA study revealed that early Xinjiang taurine cattle from the Jirentai Goukou and Xiaohe sites (∼3500 a BP) have a closer affinity to West Eurasian taurine cattle than to other East Asian taurine cattle [4]. Therefore, it is conceivable that the 3200-year-old taurine cattle at the Gepa Serul cemetery arrived at the western Tibetan Plateau via this ancient corridor.

Another important question concerns the hybridization history of cattle and yak on the Tibetan Plateau. Previous aDNA research has shown that, at least as early as 2500 a BP, yak–cattle hybridization occurred on the southern Tibetan Plateau [6]. The yak introgression facilitated the high-altitude adaptation of Tibetan cattle [10]. In this study, we used the *f*_4_ ratio and qpAdm analysis to quantify the possible introgression of yak into earlier Tibetan taurine cattle. We detected no yak introgression in KR14_4k (Fig. 1e and f, and Tables S7 and S8), whereas ZGM2_3.2k may have carried minor (∼1%) yak ancestry (Fig. 1e and f, and Tables S7 and S8). ZGM1_2k, in contrast, shared 6%–7% of its alleles with yak (Fig. 1e and f, and Tables S7 and S8). Similarly, as indicated by the *f*_4_ ratio and qpAdm analysis results (Fig. 1e and f, and Tables S7 and S8), ancient Bangga cattle (∼2500 a BP) inherited a level of yak ancestry (7.1%) comparable to that of ZGM1_2k, which is consistent with the findings of previous studies [5,6] suggesting the continuous deepening of yak–cattle hybridization on the Tibetan Plateau since 3200 a BP.

In this study, we identified three ancient taurine cattle dating to between 4000 and 2000 cal a BP recovered in archeological contexts from the eastern and western sectors of the Tibetan Plateau—two regions that are historically less investigated—and quantified their ancestries. These findings highlight that taurine cattle were introduced onto the eastern plateau before 4000 a BP, as represented by the sample from Karuo. No yak ancestry was detected in the genome of ancient Karuo cattle, indicating either that the early biogeography of yak excluded regions near Karuo or that yak domestication post-dated 4000 a BP. Our results show that yak introgression into cattle can be traced back to ≥3200 a BP, as revealed by ancient genomes from Gepa Serul, predating previous documentations by approximately seven centuries [6]. The gene flow between taurine cattle and yak intensified over the subsequent millennium. The limited sample size and dataset resolution in this study make the identification of environmentally adaptive loci during introgressive episodes particularly challenging; future investigations with broader sampling will therefore be both timely and plausible. Additionally, this work reveals two different routes for the early introduction of taurine cattle onto the Tibetan Plateau: an eastern route through the northeastern plateau toward the southernmost plateau no later than 4000 a BP and a western route likely via southern Xinjiang into Ngari, western Tibet no later than 3200 a BP. No indicine ancestry was detected from the three ancient cattle genomes under consideration. This resonates with previous observations in southern Tibet [6] indicating genetic isolation between taurine and indicine cattle separated by the Himalayan range. This further supports the interpretation that the individuals recovered at Gepa Serul were not derived from South Asia despite their close geographic proximity and suggests the existence of a western route of taurine cattle dispersal via southern Xinjiang or elsewhere in the Pamir Mountains—a region for which no ancient genomic data are currently available. We wish to draw scholarly attention to this open inquiry.

Both the eastern and western Tibetan Plateaus are often seen as isolated geographic ranges with high-elevation physical and environmental conditions; however, archeological studies have revealed that they were nevertheless key crossroads connecting ancient communities across East, South and Central Asia. Our results further illustrated such revised perception by showing their pivotal roles in early dispersals of taurine cattle and in the development of innovative practices for successfully adapting lowland-originated livestock to high-altitude environments, as well as the role of such practice in deep-time globalization.

## Supplementary Material

nwag201_Supplemental_File

## Data Availability

The DNA sequences reported in this paper have been deposited in the Genome Sequence Archive (GSA) in the National Genomics Data Center under accession PRJCA050301.
