# Supplementary material for: Ancient genomic evidence reveals dual migration routes and yak introgression history of taurine cattle on the Tibetan Plateau
Source: Natl Sci Rev. 2026 Mar 31;13(8):nwag201. doi: 10.1093/nsr/nwag201 (PMC13137984; doi:10.1093/nsr/nwag201)
Supplement: nwag201_Supplemental_File [file nwag201_supplemental_file.docx]

**Ancient genomic evidence reveals dual migration routes and yak introgression history of taurine cattle on the Tibetan Plateau**

Shungang Chen^1,2^, Jiawen Hou^3,4^, Yu Gao^2^, Guilian Sheng^5,6^, Ying Zhang^7^, Shargan Wangdue^8^, Zeba Duoji^9^, Songmei Hu^10,11^, Qiaomei Fu^12,13^, Xinyi Liu^14^, Ningbo Chen^15,^*, Fahu Chen^1,2,^*

^1^Key Laboratory of Western China’s Environmental Systems (Ministry of Education), College of Earth and Environmental Sciences, Lanzhou University, China;

^2^Alpine Paleoecology and Human Adaptation Group (ALPHA), State Key Laboratory of Tibetan Plateau Earth System, Environment and Resources (TPESER), Institute of Tibetan Plateau Research, Chinese Academy of Sciences, China;

^3^Key Laboratory of Genetic Evolution & Animal Models, Kunming Institute of Zoology, Chinese Academy of Sciences, China;

^4^Yunnan Key Laboratory of Integrative Anthropology, Kunming Institute of Zoology, Chinese Academy of Sciences, China;

^5^School of Environmental Studies, China University of Geosciences (Wuhan), China;

^6^State Key Laboratory of Geomicrobiology and Environmental Changes, China University of Geosciences (Wuhan), China;

^7^National Centre for Archaeology, China;

^8^Tibetan Institute of the Preservation of Cultural Relics, China;

^9^Changdu Institute of the Preservation of Cultural Relics, China;

^10^Key Laboratory of Archaeological Sciences and Technology (Ministry of Education), Shandong University, China;

^11^Institute of Cultural Heritage, Shandong University, China;

^12^Key Laboratory of Vertebrate Evolution and Human Origins, Institute of Vertebrate Paleontology and Paleoanthropology, Chinese Academy of Sciences, China;

^13^University of the Chinese Academy of Sciences, China;

^14^Department of Anthropology, Washington University in St. Louis, USA;

^15^Key Laboratory of Animal Genetics, Breeding and Reproduction of Shaanxi Province, College of Animal Science and Technology, Northwest A&F University, China

*** **Corresponding authors.** Emails: fhchen@itpcas.ac.cn; ningbochen@nwafu.edu.cn

**Supplementary Information**

*Extraction and library preparation of ancient DNA*

We collected three bovine samples from Karuo site and Gepa Serul cemetery on the Tibetan Plateau (Fig. 1a, Fig. S1, and Table S1). The samples were processed in the dedicated ancient DNA (aDNA) laboratory at the China University of Geoscience (Wuhan). DNA extraction was performed following established protocols [1]. The samples were subjected to UV light decontamination for 30 min on each side and surface cleaning using a sterile dentistry drill. Approximately 100 mg of powder was collected for each extraction, and DNA contamination was removed using 5% sodium hypochlorite [2]. The sample powder was incubated in lysis buffer (0.5 M EDTA, pH 8.0; water; Tween 20; and proteinase K at a concentration of 10 mg/ml) for 24 h at 37 °C [1]. DNA binding was performed by adding binding buffer D (composed of guanidine hydrochloride at a concentration of 5 M, isopropanol at a concentration of 40%, sodium acetate at a concentration of 0.12 M, and Tween 20 at a concentration of 0.05%), followed by purification using silica spin columns [1]. The extracted DNA was quantified using a Qubit^TM^ dsDNA HS Assay Kit from Thermo Fisher Scientific, and its quality was assessed using a High Sensitivity DNA Kit from Agilent Technologies.

Double-stranded libraries were prepared for these three samples using the NEBNext Ultra II DNA Library Prep Kit for Illumina (New England Biolabs) and KAPA Dual-Indexed Adapter Kit (KAPA), following the manufacturers’ protocols [3]. PCR was performed using a KAPA HiFi Uracil + Kit and KAPA Library Amplification Primer Premix. Subsequently, the PCR products were purified by size selection utilizing beads. Each extraction and library preparation series included a negative control.

*Refining the sequencing and removing adapters*

The libraries of the three studied samples were sequenced using an Illumina HiSeq X Ten platform (paired-end 150 bp, PE-150). All the raw reads were subjected to adapter trimming, and low-quality reads were removed. Furthermore, merged paired ends were utilized to generate a single sequence via LeeHom software (--ancientdna) [4].

*Generation of mitochondrial DNA sequences and subsequent phylogenetic analyses*

We utilized the Burrows–Wheeler Alignment Tool (BWA v0.7.5ar405) [5] with subcommands (aln (-l 1024 and -n 0.01) and samse (-r)) to align our trimmed reads with the mitogenome references of taurine cattle (GenBank accession number V00654.1). This process generated unfiltered SAM files that were subsequently converted into BAM format using SAMtools (v0.1.19) [6] with the view subcommand (-Sb). The resulting BAM files were sorted using the sort subcommand to eliminate PCR duplicates (MarkDuplicates REMOVE_DUPLICATES = true), and BAM files for each sample were merged using Picard tools (v2.20.3, http://broadinstitute.github.io/picard/). Subsequently, indel realignment was performed utilizing the Genome Analysis Tool Kit (GATK; v3.8-1-0-gf15c1c3ef) [7]. The reads that were unaligned or had a mapping quality less than 25 were filtered out using SAMtools (view -F 4 -b -q 25) [6]. Finally, reads shorter than 30 bp were removed from further analysis using SAMtools (-e 'length(seq)>=30') [6]. Three genomes were successfully aligned to the taurine mitogenome (GenBank accession number V00654.1). The final mtDNA BAM files were assembled with Mapping Iterative Assembler v1.0 [8], followed by manual inspection and visual verification of all the mutations against the reference sequence.

The species were identified by aligning the mitogenomes of yak, aurochs, taurine and indicine cattle (Table S2). Newly assembled sequences in this study were aligned using MUSCLE (v3.8.31) [9]. Phylogenetic relationships were inferred using the maximum likelihood (ML) approach within IQ-TREE [10]. The best-fit model determined by ModelFinder was TIM2+F+R3 [11]. Bootstrap support values for the ML analysis were generated with 1,000 replicates (-bb 1000) (Fig. 1b).

*Alignment of ancient and modern sequencing reads to the reference genome of cattle*

The ancient samples (Table S4) were aligned to the BosTau9 reference genome (ARS-UCD1.2), which included the Y chromosome from BosTau7. Alignments were performed using the BWA Tool (v0.7.5ar405) [5] with subcommands (aln (-l 1024 and -n 0.01) and samse (-r)) for defining the read group-filtered SAM files. Subsequently, the SAM files were converted to the BAM format using SAMtools (v0.1.19) [6] with the subcommand view (-Sb), followed by sorting using the subcommand sort.

PCR duplicates were removed (MarkDuplicates REMOVE_DUPLICATES = true), and the BAM files were merged using Picard tools (v2.20.3, http://broadinstitute.github.io/picard/). Indel realignment was further conducted using GATK [7]. Reads that remained unaligned or had a mapping quality less than 25 were filtered out using SAMtools (view -F 4 -b -q 25) [6]. Finally, reads shorter than 30 bp in length were discarded via SAMtools (-e 'length(seq)>=30') [6].

The mapping quality and coverage of the alignment data were assessed using Qualimap v2.2.1 (Table S4) [12]. The damage patterns of aDNA were characterized utilizing mapDamage v2.1.1 (Fig. S3) [13]. To mitigate the impact of deamination on downstream analysis, we rescaled and trimmed the DNA base damage parameters estimated with mapDamage v2.2.1 [13].

The quality-filtered reads from extant cattle, yak, and buffalo genomes (Table S3) were aligned to the ARS-UCD1.2 plus Y chromosome from BosTau7 using the BWA subcommand mem [5] and converted into BAM files using the SAMtools subcommand view (-Sb) [6]. Duplicated reads were filtered out using Picard tools (v2.20.3, http://broadinstitute.github.io/picard/), and GATK [7] was used for realigning reads around indels.

*Sex determination*

We used SAMtools idxstats [14] to quantify the reads aligned to the X chromosome and autosomes. Subsequently, a Python code for sex determination [15] was used to determine the genetic sex of the three samples (Fig. S4).

*Identification and genotyping of* *genomic variants*

The BCFtools v1.9 [6] mpileup and call commands were used to perform variant calling on the extant sample set (-q 30-C 50-Q 20-B-a FORMAT/AD, FORMAT/SP, FORMAT/ADF, FORMAT/ADR) and to generate compressed variant call format (vcf) files (-O z-m-o). Indels and variants within 3 bp of indels were filtered out using the BCFtools filter [6]. Repeat regions defined by the UCSC Browser and RepeatMasker files were excluded from the analysis [16]. Triallelic and quad-allelic sites were removed. Variants with coverage greater than three times the mean or less than one-third of the mean coverage were marked as missing (“./.”). Individuals with heterozygous variants present in only one sample or more than 75% of samples were discarded. Variant positions with missing data for 90% of the samples were ultimately eliminated. Finally, linkage disequilibrium (LD) pruning was performed using PLINK v1.9 [17] with the parameters --indep-pairwise 50 5 0.1.

The extant sites defined above were called for ancient samples with coverage greater than twice the mean using the BCFtools mpileup without both recalibration (BCFtools mpileup -B) and filtering for variant sites with BCFtools [6]. Indels and sites within 3 bp of the indels and three or four allelic sites were removed. The sites with more than twice the mean coverage were considered missing, as described earlier. To pseudodiploidize individuals, random sampling was used to obtain a read at each site, and the samples were assigned a homozygous state for the corresponding allele. Sites were set to be missing more than twice the mean coverage. For samples with depths less than twice the mean, no minimum coverage filter was applied, allowing a maximum of four reads per site.

Finally, the extant and ancient call sets were merged, and variants not present in any ancient samples were excluded. Triallelic and quad-allelic sites were also eliminated. To mitigate the impact of residual DNA damage, transitions were disregarded in subsequent analyses. Consequently, the final call set comprised 3,072,970 SNPs.

*Principal component analysis*

We conducted principal component analysis (PCA) by incorporating three ancient samples in this study along with the aurochs and ancient taurine genomes alongside extant taurine and indicine genomes from Eurasia. We computed genotype likelihoods (GLs) in ANGSD v0.921 [18] for all individuals specifying the following parameters: minimum mapping and base qualities of 30 (-minmapQ 30 -minQ 30); calculated genotype likelihoods using the GATK algorithm (-GL 2); output a beagle GL file (-doGlf 2); calculated major and minor alleles based on GL (-doMajorMinor 1); removed transitions (-rmtrans 1); included only SNPs with a *P* value <1e-6 (-SNP_pval 1e-6); considered only autosomal chromosomes (-rf), a minimum minor allele frequency of 0.05 (-minmaf 0.05); skipped triallelic sites (-skiptriallelic 1); and considered only reads mapping to one region uniquely (-uniqueonly 1). We used PCAngsd v0.98 [19] to construct covariance matrices from the GL datasets involving 1,216,474 SNPs (Table S5) for PCA (Fig. 1c).

*Identity-by-state analysis*

To develop an alternative approach to visually assess the relationship between aurochs and domestic cattle, we constructed an identity-by-state matrix using ANGSD (v0.921; -minMapQ 30 -minQ 20 -GL 1 -doMajorMinor 1 -doMaf 1 -SNP_pval 1e-6 -doGlf 2 -rmtrans 1 -skipTriallelic 1, -minMaf 2/2 N, -minFre 1/N) [18]. We used the sample with the greatest depth of sequencing in each ancient population, along with extant cattle in Eurasia (Fig. 1d).

*Admixture analysis*

An admixture analysis of aurochs and cattle on the basis of genotype likelihoods was conducted using NGSadmix [20]. The genotype likelihoods were extracted using ANGSD (v0.921; -minMapQ 30 -minQ 20 -GL 1 -doMajorMinor 1 - doMaf 1 -SNP_pval 1e-6 -doGlf 2 -rmtrans 1 -skipTriallelic 1) [18], followed by running NGSadmix (- minMaf 1/2 N) across a range of *K* values from 2-4 (Fig. S5). The optimal value of *K* was determined through iterative runs of NGSadmix as described above.

*D statistics*

*D* statistics (ABBA-BABA) were used to investigate allele sharing between ancient taurine genomes by using ANGSD v0.921 (-doAbbababa 2) [18]. Analysis was restricted to sites with base quality and mapping quality above 20 (-minQ 20 -minmapq 20) and transversions (-rmTrans 1), using a water buffalo (*Bubalus bubalis*) as an outgroup and applying the following parameters: -doCounts 1 -useLast 1 -blockSize 1000000. Only *D* statistics with a *Z* score above 3 and below -3 were considered significant (Fig. S6 and Table S6).

*qpGraph analysis*

A heuristic search of the qpGraph space using qpBrute [21] were conducted to iteratively fit complex admixture models. Starting from the root, at each iteration, a new leaf was added to the graph until all the populations included in that run were exhausted. If inserting a node either directly on a branch or as an outcome of an admixture event would result in *f*_4_ outliers, the subgraph was discarded. In each run, the included populations represented merged clusters of *Bos* individuals rooted in water buffaloes (Fig. S7).

*The f_4_ ratio and qpAdm modeling*

The qpF4 ratio software in ADMIXTOOLS [22] was used to estimate the introgression from yak to ancient Tibetan cattle (KR14_4k, ZGM1_2k, ZGM2_3.2k, and BG-33), with water buffalo serving as the outgroup (Fig. 1e and Table S7).

The admixture model for ancient Tibetan cattle (KR14_4k, ZGM1_2k, ZGM2_3.2k, and BG-33) ancestry was calculated using qpAdm from ADMIXTOOLS [22]. The “allsnps: YES” option was employed to calculate each *f*_4_ statistic (Fig. 1f and Table S8). The ancient Tibetan cattle (KR14_4k, ZGM1_2k, ZGM2_3.2k, and BG-33) and yak were considered target and source populations, respectively, whereas water buffalo, ancient and modern Eurasian taurine cattle were included as reference populations [23].

**References**

1. Rohland N, Glocke I, Aximu-Petri A, et al. Extraction of highly degraded DNA from ancient bones, teeth and sediments for high-throughput sequencing. Nat Protoc 2018; 13: 2447-61.
2. Petra K, Tobias G, Marie-Theres G, et al. Reducing microbial and human contamination in DNA extractions from ancient bones and teeth. BioTechniques 2015; 59: 87-93.
3. Meyer M, Kircher M. Illumina sequencing library preparation for highly multiplexed target capture and sequencing. Cold Spring Harb Protoc 2010; pdb. prot5448.
4. Gabriel R, Udo S, Janet K. leeHom: adaptor trimming and merging for Illumina sequencing reads. Nucleic Acids Res 2014; 42: e141.
5. Li H, Durbin R, Fast and accurate short read alignment with Burrows–Wheeler transform. Bioinformatics 2009; 25: 1754-60.
6. Li H, Handsaker B, Wysoker A, et al. The Sequence Alignment/Map format and SAMtools. Bioinformatics 2009; 25, 2078-9.
7. McKenna A, Hanna M, Banks E, et al. The Genome Analysis Toolkit: A MapReduce framework for analyzing next-generation DNA sequencing data. Genome Res 2010; 20: 1297-1303.
8. Briggs AW, Good JM, Green RE, et al. Targeted retrieval and analysis of five Neandertal mtDNA genomes. Science 2009; 325: 318-21.
9. Robert C, Edgar. MUSCLE: multiple sequence alignment with high accuracy and high throughput. Nucleic Acids Res 2004; 32: 1792-7.
10. Lam-Tung N, Schmidt HA, Arndt VH, et al. IQ-TREE: a fast and effective stochastic algorithm for estimating maximum-likelihood phylogenies. Mol Biol Evol 2015; 32: 268-74.
11. Kalyaanamoorthy S, Minh BQ, Wong TKF, et al. ModelFinder: fast model selection for accurate phylogenetic estimates. Nat Methods 2017; 14: 587-9.
12. Fernando G, Konstantin O, José C, et al. Qualimap: evaluating next-generation sequencing alignment data. Bioinformatics 2012; 28: 2678-9.
13. Jonsson H, Ginolhac A, Schubert M, et al. mapDamage2.0: fast approximate Bayesian estimates of ancient DNA damage parameters. Bioinformatics 2013; 29: 1682-4.
14. Li H, A statistical framework for SNP calling, mutation discovery, association mapping and population genetical parameter estimation from sequencing data. Bioinformatics 2011; 27: 2987-93.
15. Gower G, Fenderson LE, Salis AT, et al. Widespread male sex bias in mammal fossil and museum collections. Proc Natl Acad Sci USA 2019; 116: 19019-24.
16. Donna K, Hinrichs AS, Furey TS, et al. The UCSC Table Browser data retrieval tool. Nucleic Acids Res 2004; 32: D493-6.
17. Purcell S, Neale B, Todd-Brown K, et al. PLINK: a tool set for whole-genome association and population-based linkage analyses. Am J Hum Genet 2007; 81: 559-75.
18. Korneliussen TS, Albrechtsen A, Nielsen R. ANGSD: Analysis of next generation sequencing data. BMC Bioinform 2014; 15: 356.
19. Meisner J, Albrechtsen A. Inferring population structure and admixture proportions in low-depth NGS data. Genetics 2018; 210: 719-31.
20. Skotte L, Korneliussen TS, Albrechtsen A. Estimating individual admixture proportions from next generation sequencing data. Genetics 2013; 195: 693-702.
21. Liu L, Bosse M, Megens H-J, et al. Genomic analysis on pygmy hog reveals extensive interbreeding during wild boar expansion. Nat Commun 2019; 10: 1992.
22. Patterson N, Price AL, Reich D. Population structure and eigenanalysis. PLoS Genet 2006; 2: e190.
23. Harney A, Patterson N, Reich D, et al. Assessing the performance of qpAdm: a statistical tool for studying population admixture. Genetics 2021; 217: iyaa045.

**Supplementary Materials**

Figs. S1-S7

Tables S1-S8


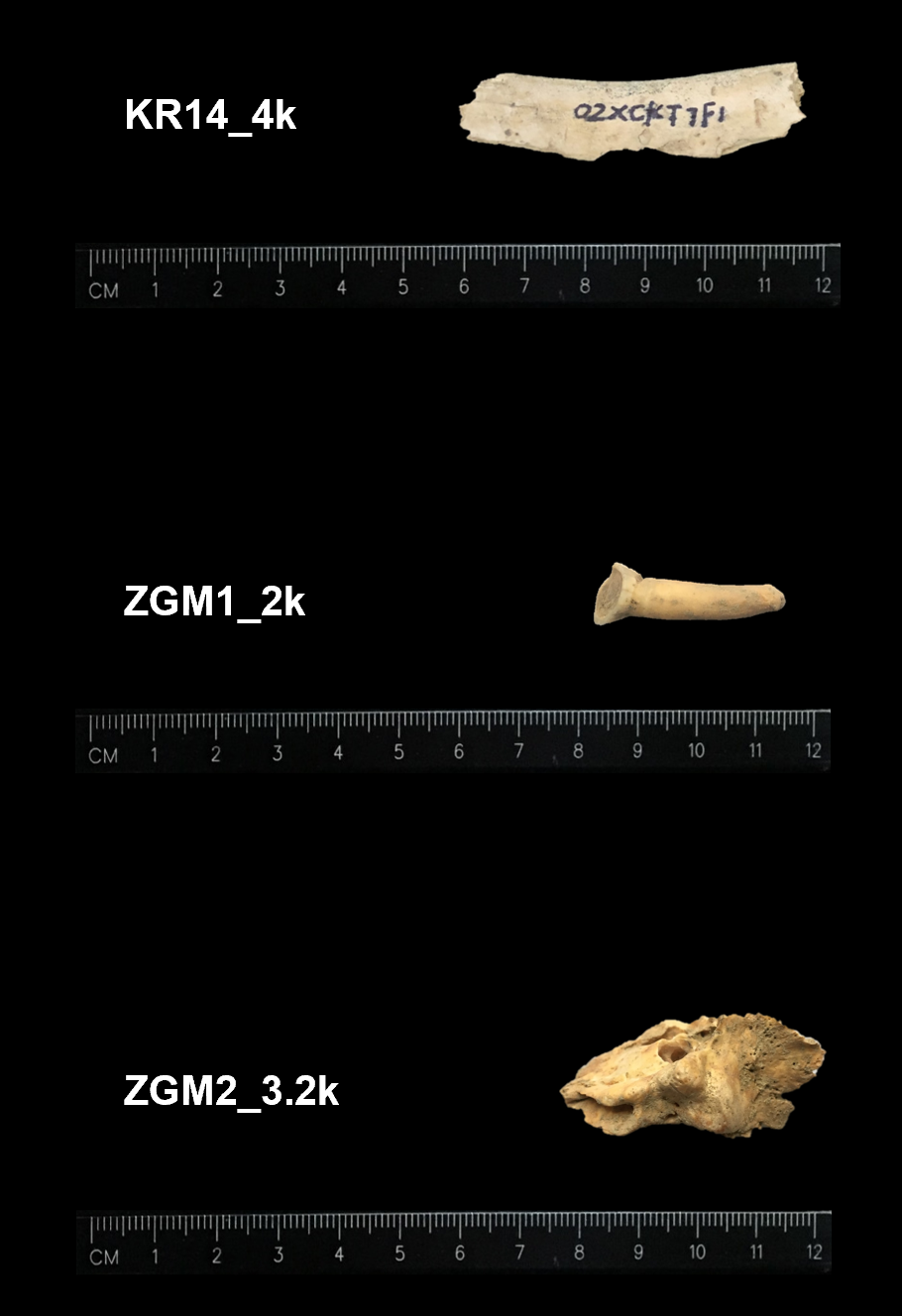


Fig. S1. Three bovine specimens analyzed in this study. KR14_4k is a broken long bone, ZGM1_2k is a tooth, and ZGM2_3.2k is a temporal bone. Images from Shungang Chen.


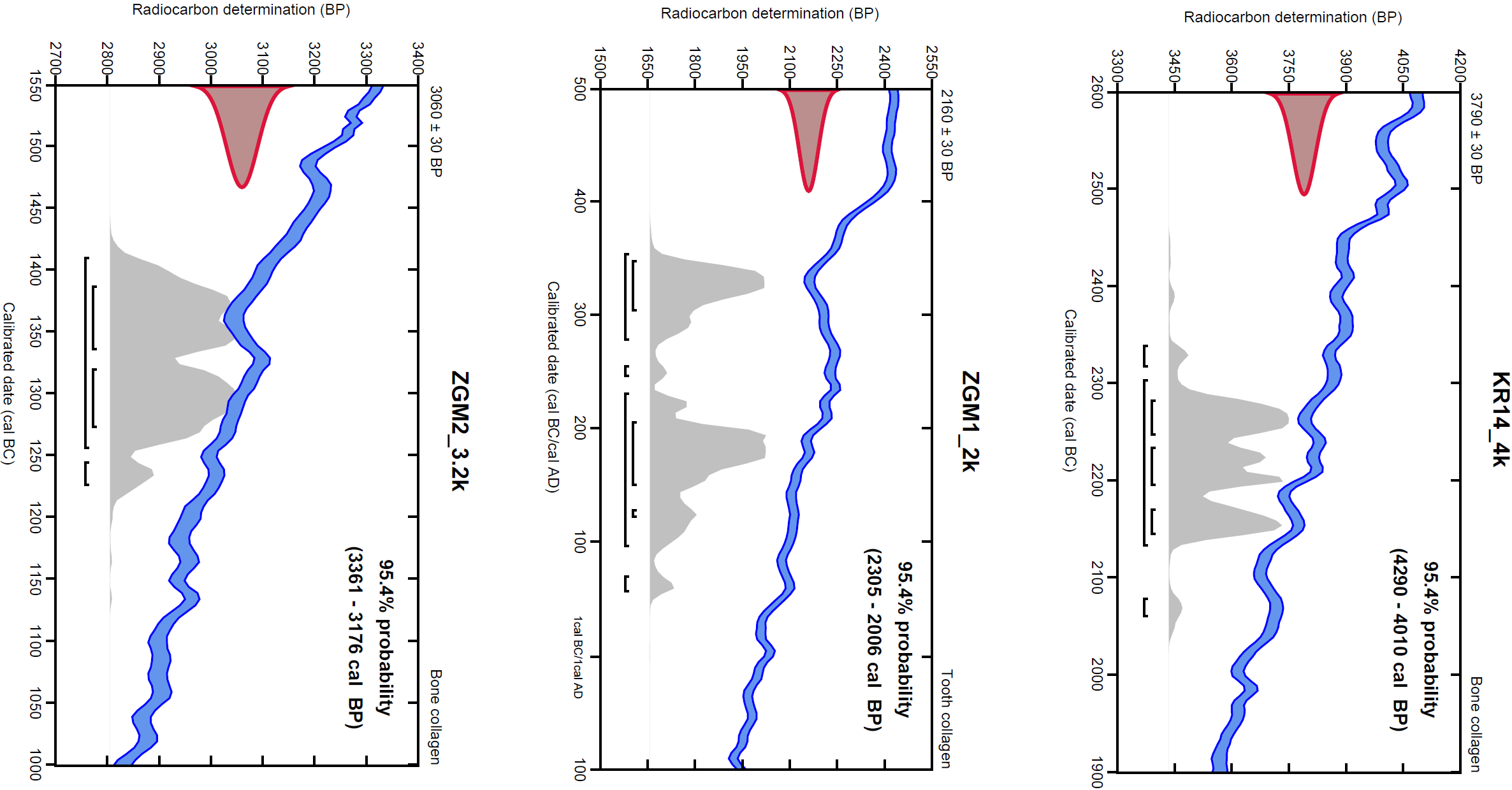


Fig. S2. Calibrated radiocarbon dating results for three bovine specimens in this study.


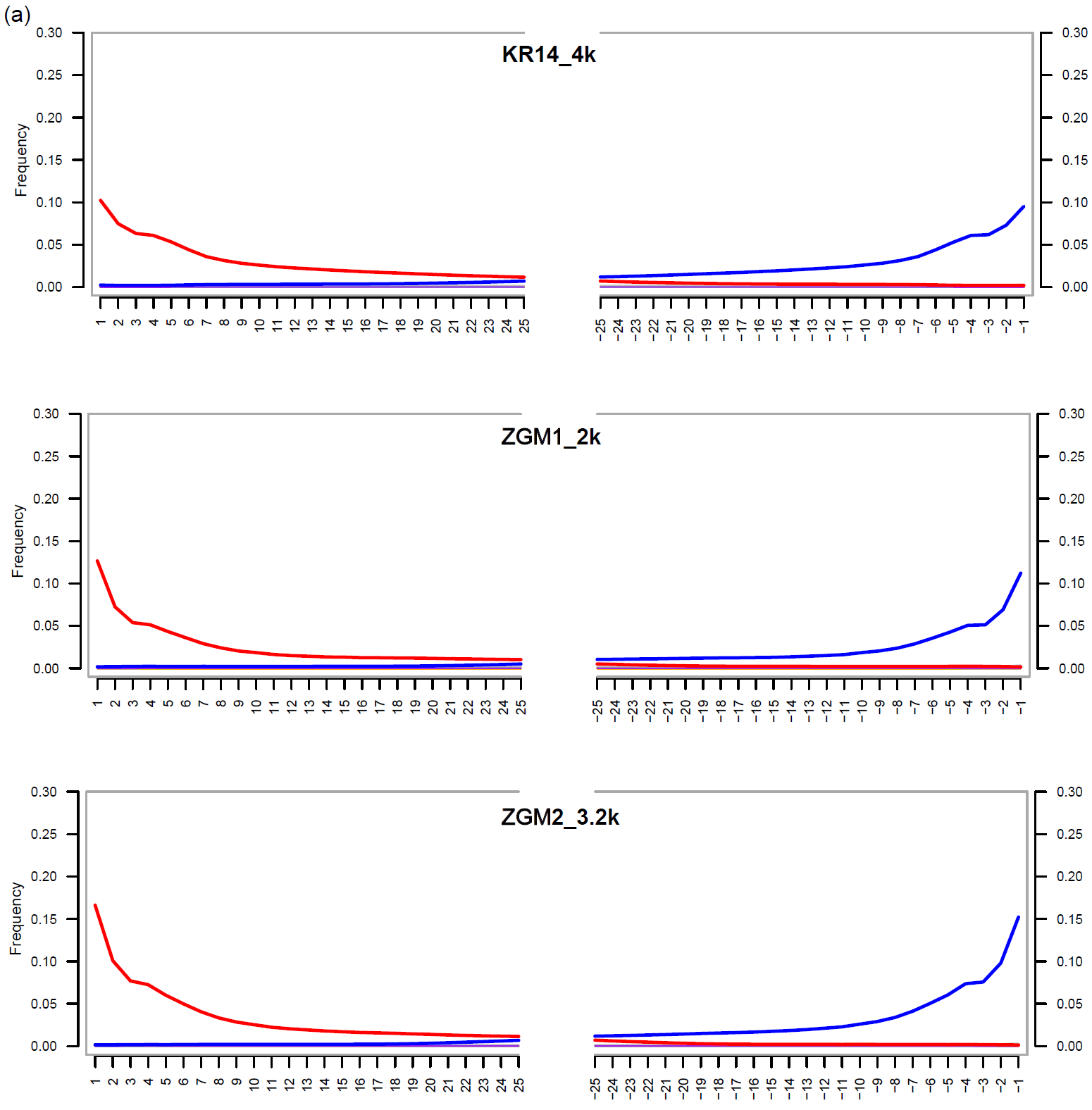


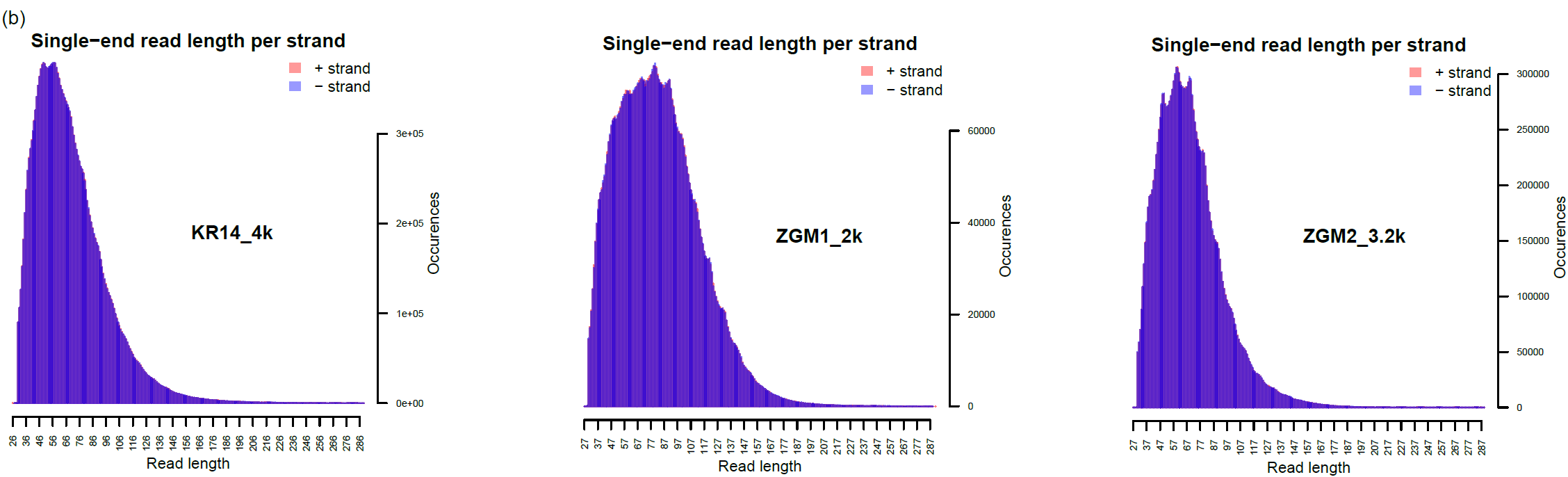


Fig. S3. Ancient DNA authentication of the three bovine samples in this study. (a) Representative terminal damage rate maps for shotgun sequences from the three bovine samples calculated by mapDamage v2.1.1. Nucleotide misincorporation patterns along the first and last 25 read positions obtained for the three samples before trimming and rescaling. The misincorporation frequencies are shown for the first and last 25 nucleotides of the reads aligned to the taurine reference nuclear genome ARS-UCD1.2. (b) aDNA fragmental length distributions of the three bovine samples plotted using their shotgun-sequenced data in alignment BAM files.


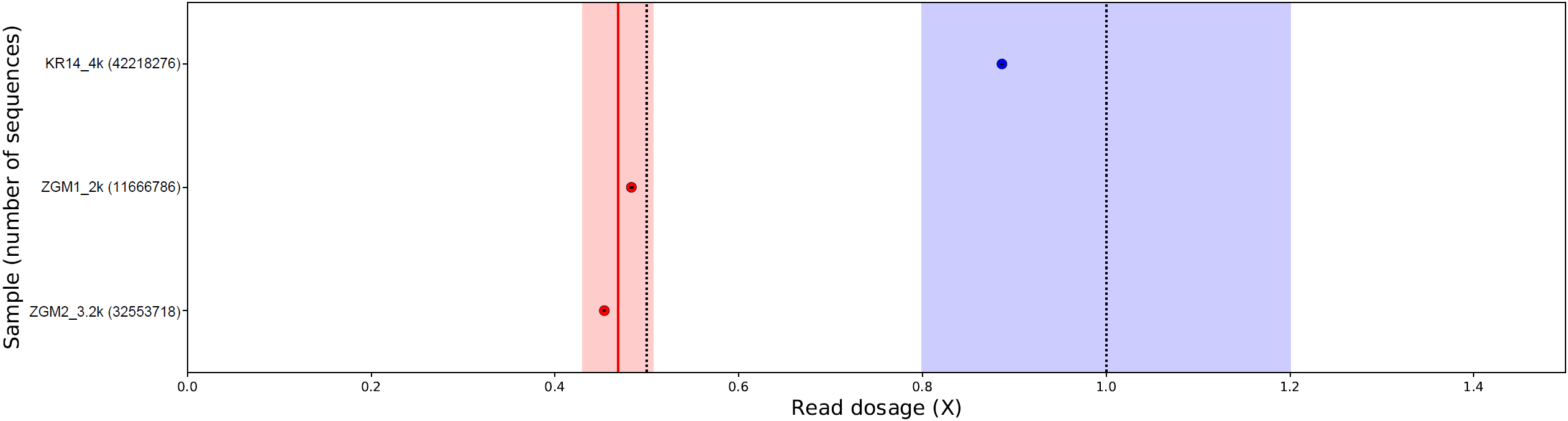


Fig. S4. Sex determination of the three bovine species in this study according to the results of the likelihood ratio test. The males cluster near 0.5, and the female clusters near 1.0.


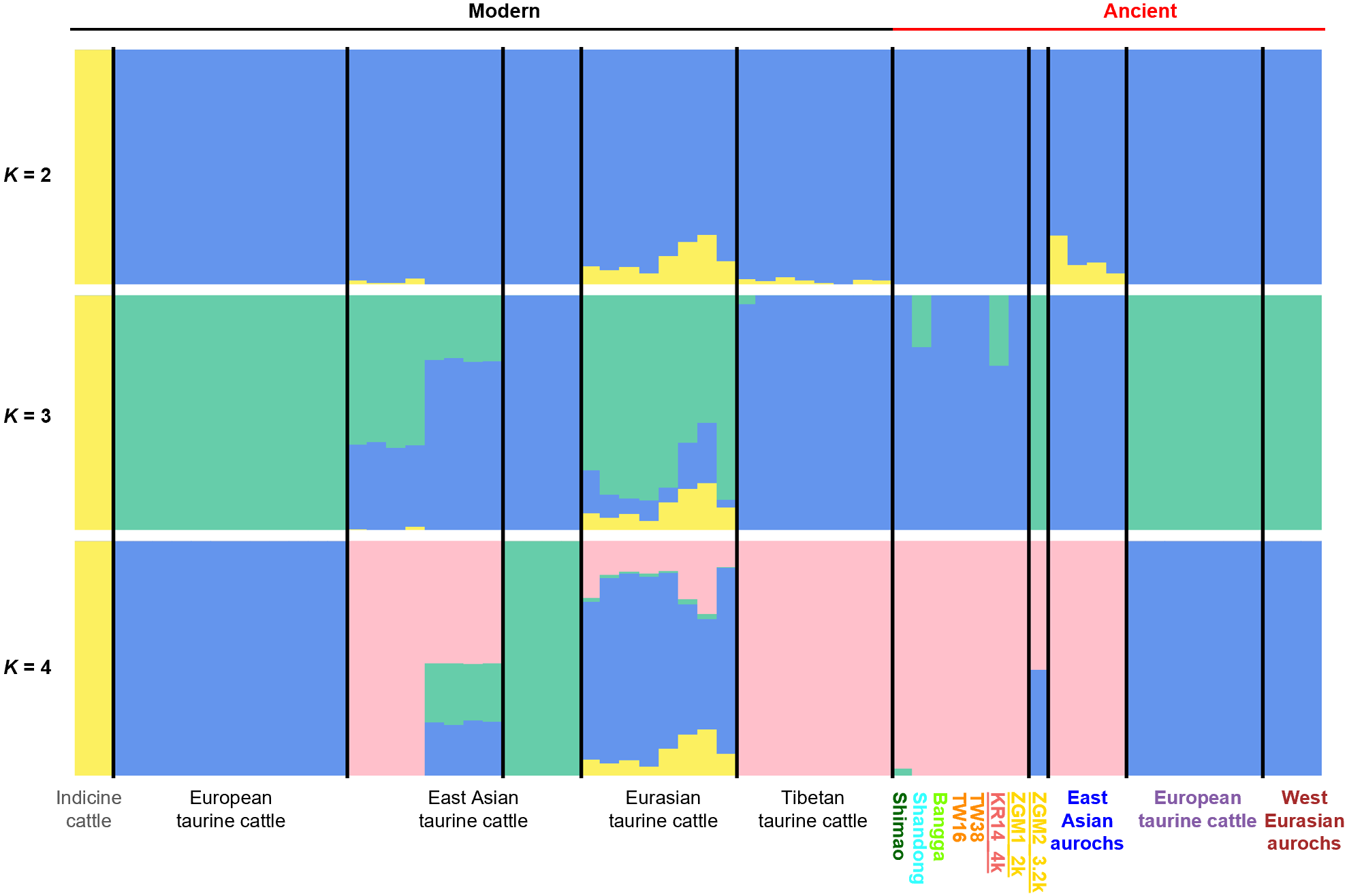


Fig. S5. Ancestry component assignment of cattle and aurochs using NGSadmix estimation with *K* = 2–4.


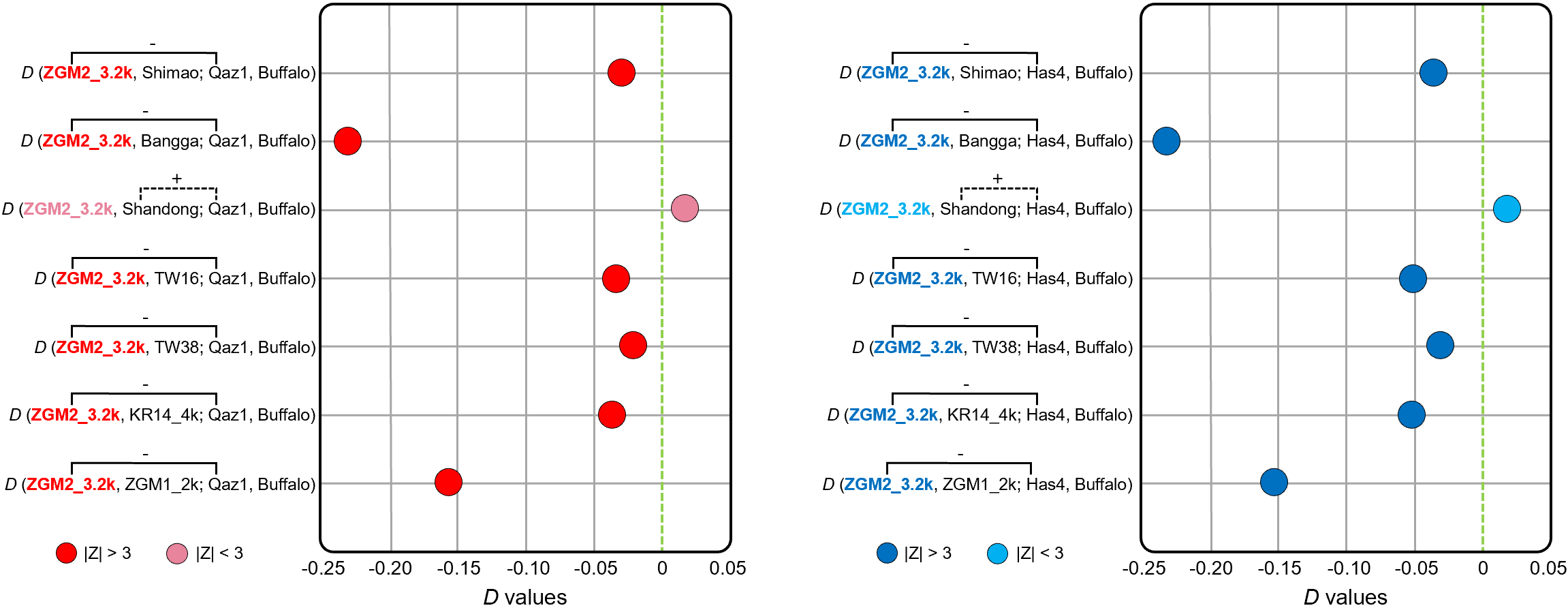


Fig. S6. Results of the *D* statistics estimated using ANGSD software with buffaloes as the outgroup. Light-colored circles indicate statistically non-significant *D* values (|*Z*| < 3).


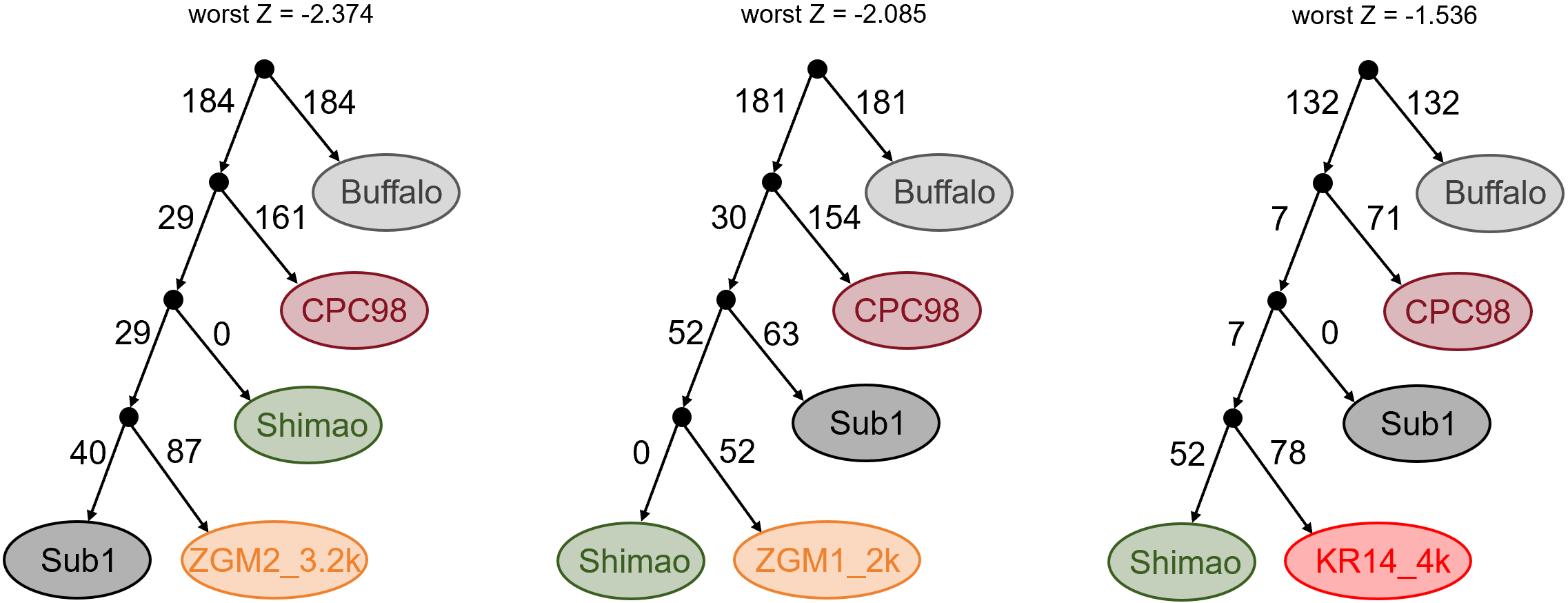


Fig. S7. Basal graph modeling using qpGraph to depict the relationships among ancient taurine cattle from different geographic regions, with water buffalo as the outgroup.

Table S1. Mapping results after merging the shotgun sequence data for three ancient bovine samples in this study.

| Sample  ID | Calibrated date (cal. BP) | Endogenous DNA | mtDNA coverage (×) | Genome coverage (×) | mtDNA identification | Genome identification |
| --- | --- | --- | --- | --- | --- | --- |
| KR14_4k | 4290-4010 | 10% | 560.70 | 1.07 | Taurine cattle | Taurine cattle |
| ZGM1_2k | 2305-2006 | 5% | 65.06 | 0.35 | Taurine cattle | Taurine cattle |
| ZGM2_3.2k | 3361-3176 | 25% | 94.60 | 0.82 | Taurine cattle | Taurine cattle |

Table S2. Overview of sample information for the mitogenomes used in this study.

| Number | Sample | Species | Haplogroup | Data source | Reference |
| --- | --- | --- | --- | --- | --- |
| 1 | Ancient01 | *Bos taurus taurus* | T4 | MT576706 | [1,3,5,6,10] |
| 2 | Ancient05 | *B. t. taurus* | T4 | MT576708 |  |
| 3 | Xizang19 | *B. t. taurus* | T4 | SRR5507245 |  |
| 4 | YB6 | *B. t. taurus* | T4 | MT576844 |  |
| 5 | DJ41 | *B. t. taurus* | T4 | MT576741 |  |
| 6 | MG1 | *B. t. taurus* | T4 | MT576769 |  |
| 7 | WMG1 | *B. t. taurus* | T4 | MT576820 |  |
| 8 | Mishima02 | *B. t. taurus* | T4 | DRR001763 |  |
| 9 | Korean | *B. t. taurus* | T4 | NC_006853 |  |
| 10 | JBC1 | *B. t. taurus* | T4 | AB074962 |  |
| 11 | Ancient04 | *B. t. taurus* | T3_055_ | MT576705 |  |
| 12 | DJ49 | *B. t. taurus* | T3_055_ | MT576748 |  |
| 13 | Xizang22 | *B. t. taurus* | T3_055_ | SRR5507249 |  |
| 14 | WMG20 | *B. t. taurus* | T3_055_ | MT576829 |  |
| 15 | KC6 | *B. t. taurus* | T3_055_ | DQ124376 |  |
| 16 | JBC6 | *B. t. taurus* | T3_055_ | AB074966 |  |
| 17 | Ancient02 | *B. t. taurus* | T3_119_ | MT576707 |  |
| 18 | Ancient07 | *B. t. taurus* | T3_119_ | MT576709 |  |
| 19 | Ancient08 | *B. t. taurus* | T3_119_ | MT576710 |  |
| 20 | BG-1 | *B. t. taurus* | T3_119_ | SRR17245064 |  |
| 21 | BG-14 | *B. t. taurus* | T3_119_ | SRR17245119 |  |
| 22 | RK21 | *B. t. taurus* | T3_119_ | MT576794 |  |
| 23 | RK27 | *B. t. taurus* | T3_119_ | MT576798 |  |
| 24 | DJ26 | *B. t. taurus* | T3_119_ | MT576729 |  |
| 25 | DJ27 | *B. t. taurus* | T3_119_ | MT576730 |  |
| 26 | BG-15 | *B. t. taurus* | T3 | SRR17245110 |  |
| 27 | BG-33 | *B. t. taurus* | T3 | SRR21608681 |  |
| 28 | H20 | *B. t. taurus* | T3 | PRJCA046277 |  |
| 29 | TW16 | *B. t. taurus* | T3 | PRJCA024258 |  |
| 30 | RK25 | *B. t. taurus* | T3 | MT576795 |  |
| 31 | DJ35 | *B. t. taurus* | T3 | MT576735 |  |
| 32 | DJ50 | *B. t. taurus* | T3 | MT576749 |  |
| 33 | DJ52 | *B. t. taurus* | T3 | MT576751 |  |
| 34 | MG35 | *B. t. taurus* | T2 | SRR5507276 |  |
| 35 | AX1909 | *B. t. taurus* | T2 | MT576714 |  |
| 36 | HSK25 | *B. t. taurus* | T2 | SRR5507260 |  |
| 37 | RKZ5 | *B. t. taurus* | T2 | MT576796 |  |
| 38 | YB4 | *B. t. taurus* | T1 | MT576842 |  |
| 39 | YB5 | *B. t. taurus* | T1 | MT576843 |  |
| 40 | HSK1 | *B. t. taurus* | T1 | SRR5507257 |  |
| 41 | A83 | *B. t. taurus* | T1 | SRR5507273 |  |
| 42 | RK17 | *B. t. taurus* | Q | MT576790 |  |
| 43 | DJ42 | *B. t. taurus* | Q | MT576742 |  |
| 44 | Gyu2 | *Bos primigenius* | Q | ERR3317399 |  |
| 45 | CPC98 | *B. primigenius* | P | GU985279 |  |
| 46 | BP01 | *B. primigenius* | P | JQ437479 |  |
| 47 | Kongni | *B. primigenius* | C | KF525852 |  |
| 48 | TW35 | *B. primigenius* | C | PRJCA024258 |  |
| 49 | TW38 | *B. primigenius* | C | PRJCA024258 |  |
| 50 | Y5 | *B. primigenius* | C | PRJNA781671 |  |
| 51 | HSK8 | *Bos taurus indicus* | I | SRR5507256 |  |
| 52 | CT13 | *B. t. indicus* | I | MT576723 |  |
| 53 | X15 | *Bos grunniens* | Yak | MW414157 |  |
| 54 | BG008 | *B. grunniens* | Yak | NC_006380 |  |
| 55 | BG-67 | *B. grunniens* | Yak | SRR21608679 |  |
| 56 | Outgroup | *Bubalus bubalis* | Buffalo | OR766451 |  |

Table S3. Information on the present-day cattle, yak and water buffalo samples used in this study.

| Number | Sample | Breeds | Mapping rate | Mean depth | Group | Species | Biosample ID | Data source | Reference |
| --- | --- | --- | --- | --- | --- | --- | --- | --- | --- |
| 1 | ERR2734942 | Finncattle | 99.84% | 10.98 | Europe | *B. taurus* | SAMEA4827182 | PRJEB28185 | [3,6,10] |
| 2 | ERR2734943 | Finncattle | 99.85% | 10.9 | Europe | *B. taurus* | SAMEA4827183 | PRJEB28185 |  |
| 3 | ERR2734944 | Finncattle | 99.84% | 10.54 | Europe | *B. taurus* | SAMEA4827184 | PRJEB28185 |  |
| 4 | ERR2734945 | Finncattle | 99.85% | 11.06 | Europe | *B. taurus* | SAMEA4827185 | PRJEB28185 |  |
| 5 | SRR1365124 | Hereford | 99.13% | 14.95 | Europe | *B. taurus* | SAMN02843135 | PRJNA176557 |  |
| 6 | SRR1365126 | Hereford | 99.32% | 15.77 | Europe | *B. taurus* | SAMN02843134 | PRJNA176557 |  |
| 7 | SRR1365128 | Hereford | 99.30% | 15.2 | Europe | *B. taurus* | SAMN02843132 | PRJNA176557 |  |
| 8 | SRR1365131 | Hereford | 99.20% | 15.31 | Europe | *B. taurus* | SAMN02843133 | PRJNA176557 |  |
| 9 | SRR1365137 | Hereford | 98.95% | 12.07 | Europe | *B. taurus* | SAMN02843090 | PRJNA176557 |  |
| 10 | SRR3497451 | Jersey | 99.85% | 12.88 | Europe | *B. taurus* | SAMN04978252 | PRJNA318089 |  |
| 11 | SRR3497464 | Jersey | 99.88% | 12.29 | Europe | *B. taurus* | SAMN04978254 | PRJNA318089 |  |
| 12 | SRR3497466 | Jersey | 99.85% | 14.8 | Europe | *B. taurus* | SAMN04978256 | PRJNA318089 |  |
| 13 | SRR3497611 | Jersey | 99.71% | 13.98 | Europe | *B. taurus* | SAMN04978258 | PRJNA318089 |  |
| 14 | SRR1525701 | Simmental | 99.02% | 12.41 | Europe | *B. taurus* | SAMN02941222 | PRJNA256210 |  |
| 15 | SRR1525702 | Simmental | 99.35% | 13.56 | Europe | *B. taurus* | SAMN02941229 | PRJNA256210 |  |
| 16 | SRR1525703 | Simmental | 94.27% | 15.17 | Europe | *B. taurus* | SAMN02941225 | PRJNA256210 |  |
| 17 | SRR1525705 | Simmental | 98.93% | 13.68 | Europe | *B. taurus* | SAMN02941199 | PRJNA256210 |  |
| 18 | MG12 | Mongolia | 99.75% | 12.25 | Eurasia | *B. taurus* | SAMN06698981 | PRJNA379859 |  |
| 19 | MG14 | Mongolia | 99.79% | 11.59 | Eurasia | *B. taurus* | SAMN06698982 | PRJNA379859 |  |
| 20 | MG17 | Mongolia | 99.81% | 11.66 | Eurasia | *B. taurus* | SAMN06698983 | PRJNA379859 |  |
| 21 | MG35 | Mongolia | 99.80% | 11.72 | Eurasia | *B. taurus* | SAMN06698984 | PRJNA379859 |  |
| 22 | HSK1 | Kazakh | 99.82% | 11.03 | Eurasia | *B. taurus* | SAMN06698989 | PRJNA379859 |  |
| 23 | HSK25 | Kazakh | 99.82% | 12.68 | Eurasia | *B. taurus* | SAMN06698986 | PRJNA379859 |  |
| 24 | HSK26 | Kazakh | 99.73% | 10.14 | Eurasia | *B. taurus* | SAMN06698991 | PRJNA379859 |  |
| 25 | HSK29 | Kazakh | 99.84% | 9.81 | Eurasia | *B. taurus* | SAMN06698992 | PRJNA379859 |  |
| 26 | SRR934415 | Hanwoo | 99.85% | 12.99 | East Asia | *B. taurus* | SAMN02225744 | PRJNA210519 |  |
| 27 | SRR934417 | Hanwoo | 99.82% | 12.77 | East Asia | *B. taurus* | SAMN02225746 | PRJNA210519 |  |
| 28 | SRR934418 | Hanwoo | 99.81% | 13.28 | East Asia | *B. taurus* | SAMN02225747 | PRJNA210519 |  |
| 29 | SRR934419 | Hanwoo | 99.82% | 12.56 | East Asia | *B. taurus* | SAMN02225748 | PRJNA210519 |  |
| 30 | ERR2734952 | Yakutian | 99.82% | 10.75 | East Asia | *B. taurus* | SAMEA4827192 | PRJEB28185 |  |
| 31 | ERR2734953 | Yakutian | 99.81% | 10.57 | East Asia | *B. taurus* | SAMEA4827193 | PRJEB28185 |  |
| 32 | ERR2734954 | Yakutian | 99.80% | 10.32 | East Asia | *B. taurus* | SAMEA4827194 | PRJEB28185 |  |
| 33 | ERR2734955 | Yakutian | 99.80% | 10.63 | East Asia | *B. taurus* | SAMEA4827195 | PRJEB28185 |  |
| 34 | Mishima01 | Mishima | 99.09% | 11.62 | East Asia | *B. taurus* | SAMD00013611 | PRJDB2660 |  |
| 35 | Mishima05 | Mishima | 98.79% | 12.39 | East Asia | *B. taurus* | SAMD00013607 | PRJDB2660 |  |
| 36 | Mishima06 | Mishima | 98.92% | 13.92 | East Asia | *B. taurus* | SAMD00013612 | PRJDB2660 |  |
| 37 | Mishima07 | Mishima | 98.71% | 12.83 | East Asia | *B. taurus* | SAMD00013609 | PRJDB2660 |  |
| 38 | YSHN-M-02 | Yushu | 99.61% | 8.28 | Tibet | *B. taurus* | SAMN31882950 | PRJNA905718 |  |
| 39 | YSHN-M-03 | Yushu | 99.83% | 9.61 | Tibet | *B. taurus* | SAMN31882951 | PRJNA905718 |  |
| 40 | YSHN-M-04 | Yushu | 99.65% | 9.26 | Tibet | *B. taurus* | SAMN31882952 | PRJNA905718 |  |
| 41 | YSHN-M-05 | Yushu | 99.83% | 7.44 | Tibet | *B. taurus* | SAMN31882953 | PRJNA905718 |  |
| 42 | YSHN-M-07 | Yushu | 99.77% | 9.11 | Tibet | *B. taurus* | SAMN31882955 | PRJNA905718 |  |
| 43 | YSHN-M-08 | Yushu | 99.81% | 8.17 | Tibet | *B. taurus* | SAMN31882956 | PRJNA905718 |  |
| 44 | Xizang7 | Changdu | 98.93% | 24.56 | Tibet | *B. taurus* | SAMN06698998 | PRJNA379859 |  |
| 45 | Xizang11 | Changdu | 99.82% | 13.58 | Tibet | *B. taurus* | SAMN06699003 | PRJNA379859 |  |
| 46 | Xizang17 | Changdu | 99.78% | 10.59 | Tibet | *B. taurus* | SAMN06698995 | PRJNA379859 |  |
| 47 | Xizang19 | Changdu | 99.80% | 10.68 | Tibet | *B. taurus* | SAMN06699001 | PRJNA379859 |  |
| 48 | Xizang21 | Changdu | 99.77% | 10.86 | Tibet | *B. taurus* | SAMN06698999 | PRJNA379859 |  |
| 49 | Xizang22 | Changdu | 98.38% | 26.28 | Tibet | *B. taurus* | SAMN06698997 | PRJNA379859 |  |
| 50 | Brahman05 | Brahman | 99.53% | 13.15 | South Asia | *Bos indicus* | SAMN05216066 | PRJNA324822 |  |
| 51 | Brahman06 | Brahman | 99.61% | 15.3 | South Asia | *B. indicus* | SAMN05216067 | PRJNA324822 |  |
| 52 | SRR2059962 | Yak | 99.67% | 8.20 | Wild | *Bos mutus* | SAMN03766785 | PRJNA285834 |  |
| 53 | SRR2059963 | Yak | 99.56% | 7.05 | Wild | *B. mutus* | SAMN03766786 | PRJNA285834 |  |
| 54 | SRR2059965 | Yak | 99.55% | 7.16 | Wild | *B. mutus* | SAMN03766788 | PRJNA285834 |  |
| 55 | SRR2059966 | Yak | 98.92% | 20.93 | Wild | *B. mutus* | SAMN03766789 | PRJNA285834 |  |
| 56 | SRR2059969 | Yak | 80.71% | 16.97 | Wild | *B. mutus* | SAMN03766792 | PRJNA285834 |  |
| 57 | SRR2062306 | Yak | 99.52% | 20.16 | Wild | *B. mutus* | SAMN03766790 | PRJNA285834 |  |
| 58 | buffalo01 | Water buffalo | 98.78% | 19.86 | Buffalo | *Bubalus bubalis* | SAMN05949028 | PRJNA350833 |  |
| 59 | buffalo02 | Water buffalo | 98.51% | 18.62 | Buffalo | *B. bubalis* | SAMN05949029 | PRJNA350833 |  |

Table S4. Information on the ancient cattle, aurochs and yak samples used in this study.

| Number | Sample ID | Data (BP) | Location | Group | Mean depth | Duplication rate | Biosample ID | Data source | Reference |
| --- | --- | --- | --- | --- | --- | --- | --- | --- | --- |
| 1 | BG-33 | 2500 | China | Bangga | 1.7230 | 30.41% | SAMN30865715 | PRJNA788155 | [1,3,5,6] |
| 2 | Ancient05 | 3900 | China | Shimao | 1.3032 | 13.91% | SAMN08866422 | PRJNA379859 |  |
| 3 | H20 | 2000 | China | Shandong | 0.0248 | 1.12% | SAMC5888718 | PRJCA046277 |  |
| 4 | TW16 | 3500 | China | Tawendaliha | 0.5095 | 3.25% | SAMC3543937 | PRJCA024258 |  |
| 5 | TW38 | 3750 | China | Tawendaliha | 0.2843 | 5.08% | SAMC3436049 | PRJCA024258 |  |
| 6 | TW35 | 3400 | China | *Bos primigenius* | 1.2929 | 4.95% | SAMC3436048 | PRJCA024258 |  |
| 7 | Y5 | 3900 | China | *Bos primigenius* | 1.5742 | 44.03% | SAMN30869329 | PRJNA781671 |  |
| 8 | Y14 | 33000 | China | *Bos primigenius* | 0.0977 | 37.75% | SAMN23286465 | PRJNA781671 |  |
| 9 | Kongni | 10660 | China | *Bos primigenius* | 0.2705 | 21.55% | SAMN47934040 | PRJNA781671 |  |
| 10 | Ch22 | 7600 | Turkey | Anatolia | 0.3205 | 4.39% | SAMEA5577358 | PRJEB31621 |  |
| 11 | CPC98 | 6750 | England | *Bos primigenius* | 1.5403 | 4.59% | SAMN04028906 | PRJNA294709 |  |
| 12 | Gyu2 | 7040 | Armenia | *Bos primigenius* | 1.6974 | 7.94% | SAMEA5577362 | PRJEB31621 |  |
| 13 | Has4 | 2900 | Iran | Iran | 1.6515 | 31.99% | SAMEA5577365 | PRJEB31621 |  |
| 14 | Men1 | 8050 | Turkey | Anatolia | 3.2496 | 21.79% | SAMEA5577376 | PRJEB31621 |  |
| 15 | Men2 | 7920 | Turkey | Anatolia | 2.2590 | 32.69% | SAMEA5577377 | PRJEB31621 |  |
| 16 | Plo3 | 7025 | Serbia | Balkans | 2.6263 | 6.94% | SAMEA5577382 | PRJEB31621 |  |
| 17 | Plo4 | 7000 | Serbia | Balkans | 2.8551 | 7.58% | SAMEA5577383 | PRJEB31621 |  |
| 18 | Qaz1 | 4760 | Iran | Iran | 1.6466 | 15.41% | SAMEA5577389 | PRJEB31621 |  |
| 19 | Sub1 | 8024 | Turkey | Anatolia | 14.0105 | 30.20% | SAMEA5577393 | PRJEB31621 |  |
| 20 | Th7 | 8801 | Morocco | *Bos primigenius* | 0.1039 | 20.96% | SAMEA5577395 | PRJEB31621 |  |
| 21 | BG-67 | 2500 | China | Ancient yak | 0.0730 | 18.01% | SAMN30865723 | PRJNA788155 |  |

Table S5. Sample and SNP information used for different analyses.

| Analysis | Number of samples | Number of SNPs |
| --- | --- | --- |
| IQ-TREE of mitogenomes | 59 | 16,349 |
| PCA using ANGSD | 69 | 1,216,474 |
| Identity-by-state analysis | 69 | 5,680,125 |
| Admixture analysis | 69 | 1,668,569 |
| *f_4_* ratio test | 26 | 2,623,595 |
| qpAdm analysis for KR14_4k | 18 | 2,607,493 |
| qpAdm analysis for ZGM1_2k | 18 | 2,577,816 |
| qpAdm analysis for ZGM2_3.2k | 18 | 2,580,975 |
| qpAdm analysis for BG-33 | 18 | 2,576,465 |

Table S6. The *D* statistics results were estimated using ANGSD with water buffaloes as the outgroup.

| H1 | H2 | H3 | *D*-stat | *P* value | nABBA | nBABA | *Z* |
| --- | --- | --- | --- | --- | --- | --- | --- |
| ZGM2_3.2k | Shimao | Qaz1 | -0.0367 | 0.000000 | 57064 | 61409 | -5.6859 |
| ZGM2_3.2k | Bangga | Qaz1 | -0.2341 | 0.000000 | 56301 | 90717 | -33.8249 |
| ZGM2_3.2k | Shandong | Qaz1 | 0.0181 | 0.275506 | 1807 | 1743 | 1.0905 |
| ZGM2_3.2k | TW16 | Qaz1 | -0.0413 | 0.000000 | 43316 | 47051 | -5.6920 |
| ZGM2_3.2k | TW38 | Qaz1 | -0.0238 | 0.000999 | 28058 | 29427 | -3.2909 |
| ZGM2_3.2k | KR14_4k | Qaz1 | -0.0449 | 0.000000 | 69776 | 76330 | -6.7285 |
| ZGM2_3.2k | ZGM1_2k | Qaz1 | -0.1614 | 0.000000 | 31112 | 43091 | -20.6531 |
| ZGM2_3.2k | Shimao | Has4 | -0.0428 | 0.000000 | 53471 | 58255 | -5.9368 |
| ZGM2_3.2k | Bangga | Has4 | -0.2379 | 0.000000 | 52109 | 84642 | -32.3382 |
| ZGM2_3.2k | Shandong | Has4 | 0.0168 | 0.331527 | 1686 | 1630 | 0.9710 |
| ZGM2_3.2k | TW16 | Has4 | -0.0521 | 0.000000 | 39761 | 44130 | -7.1029 |
| ZGM2_3.2k | TW38 | Has4 | -0.0358 | 0.000004 | 25898 | 27820 | -4.6320 |
| ZGM2_3.2k | KR14_4k | Has4 | -0.0552 | 0.000000 | 63975 | 71445 | -8.1093 |
| ZGM2_3.2k | ZGM1_2k | Has4 | -0.1589 | 0.000000 | 29147 | 40159 | -19.9553 |

Table S7. Introgression results based on the *f_4_* ratio test performed using ADMIXTOOLS.

| Population A | Population B | Population X | Population C | Population O | Alpha | SE | *Z* |
| --- | --- | --- | --- | --- | --- | --- | --- |
| Sub1 | Shimao | KR14_4k | Wild yak | Buffalo | 1.001 | 0.006 | 161.781 |
| Sub1 | Shimao | ZGM1_2k | Wild yak | Buffalo | 0.939 | 0.027 | 35.020 |
| Hereford | Sub1 | ZGM2_3.2k | Wild yak | Buffalo | 0.990 | 0.010 | 100.433 |
| Sub1 | Shimao | BG-33 | Wild yak | Buffalo | 0.929 | 0.009 | 105.472 |

Table S8. Detailed qpAdm results of the two-source model.

| Sample | Source 1 | Source 1 value | Source 2 | Source 2 value | SE | *P* value |
| --- | --- | --- | --- | --- | --- | --- |
| KR14_4k | Shimao | 0.999 | Wild yak | 0.001 | 0.005 | 0.572612 |
| ZGM1_2k | Shimao | 0.930 | Wild yak | 0.070 | 0.021 | 0.567785 |
| ZGM2_3.2k | Sub1 | 0.988 | Wild yak | 0.012 | 0.013 | 0.253662 |
| BG-33 | Shimao | 0.929 | Wild yak | 0.071 | 0.007 | 0.944900 |
